# Midterm outcome and strength assessment after proximal rectus femoris refixation in athletes

**DOI:** 10.1007/s00402-021-04189-0

**Published:** 2021-10-18

**Authors:** Maximilian Hinz, Stephanie Geyer, Felix Winden, Alexander Braunsperger, Florian Kreuzpointner, Benjamin D. Kleim, Andreas B. Imhoff, Julian Mehl

**Affiliations:** 1 Department of Orthopaedic Sports Medicine, Klinikum rechts der Isar, Technical University of Munich, Ismaninger Straße 22, 81675 Munich, Germany; 2 Prevention Center, Department of Sport and Health Sciences, Technical University of Munich, Munich, Germany

**Keywords:** Quadriceps, Sports Injury, Return to competition, Soccer, Surgical treatment, Acute, Chronic

## Abstract

**Purpose:**

Proximal rectus femoris avulsions (PRFA) are relatively rare injuries that occur predominantly among young soccer players. The aim of this study was to evaluate midterm postoperative results including strength potential via standardized strength measurements after proximal rectus femoris tendon refixation. It was hypothesized that the majority of competitive athletes return to competition (RTC) after refixation of the rectus femoris tendon without significant strength or functional deficits compared to the contralateral side.

**Methods:**

Patients with an acute (< 6 weeks) PRFA who underwent surgical refixation between 2012 and 2019 with a minimum follow-up of 12 months were evaluated. The outcome measures compiled were the median Tegner Activity Scale (TAS) and mean RTC time frames, Harris Hip Score (HHS), Hip and Groin Outcome Score (HAGOS) subscales, International Hip Outcome Tool-33 (iHOT-33), and Visual Analog Scale (VAS) for pain. In addition, a standardized isometric strength assessment of knee flexion, knee extension, and hip flexion was performed to evaluate the functional result of the injured limb in comparison to the uninjured side.

**Results:**

Out of 20 patients, 16 (80%) patients were available for final assessment at a mean follow-up of 44.8 ± SD 28.9 months. All patients were male with 87.5% sustaining injuries while playing soccer. The average time interval between trauma and surgery was 18.4 ± 8.5 days. RTC was possible for 14 out of 15 previously competitive athletes (93.3%) at a mean 10.5 ± 3.4 months after trauma. Patients achieved a high level of activity postoperatively with a median (interquartile range) TAS of 9 (7–9) and reported good to excellent outcome scores (HHS: 100 (96–100); HAGOS: symptoms 94.6 (89.3–100), pain 97.5 (92.5–100), function in daily living 100 (95–100), function in sport and recreation 98.4 (87.5–100), participation in physical activities 100 (87.5–100), quality of life 83.1 ± 15.6; iHot-33: 95.1 (81.6–99.8)). No postoperative complications were reported. Range of motion, isometric knee flexion and extension, as well as hip flexion strength levels were not statistically different between the affected and contralateral legs. The majority of patients were “very satisfied” (56.3%) or “satisfied” (37.5%) with the postoperative result and reported little pain (VAS 0 (0–0.5)).

**Conclusion:**

Surgical treatment of acute PRFA yields excellent postoperative results in a young and highly active cohort. Hip flexion and knee extension strength was restored fully without major surgical complications.

**Level of evidence:**

Retrospective cohort study; III.

## Introduction

The majority of muscle injuries in soccer occur in the lower extremity, predominantly to the hamstrings, adductors and quadriceps, of which quadriceps injuries have the second-highest recurrence rate and lead to the longest time of absence in sports [12]. Within quadriceps injuries, the rectus femoris (RF) muscle is known to be the most commonly affected muscle [8, 29]. Proximal RF injuries mainly occur during sprints and kicking movements [19], and present as myofascial injuries, small to complete ruptures at the myotendinous junction, midsubstance ruptures, or avulsion injuries from its anatomic origin [4, 9, 15, 19, 21–23, 25, 26, 33]. For proximal rectus femoris avulsions (PRFA), operative treatment options include suture anchor repair and tenodesis with excision of the proximal tendon in which  both alternatives yield satisfactory results primarily in small case series [23, 26, 28, 32]. Alternatively, non-operative treatment is often chosen with high return to play (RTP) rates after 21–208 days reported [14]. However, there is still no consensus on the treatment for PRFA in athletes who participate in physically demanding sports, the cohort that is predominantly affected by this injury.

For surgical treatment, both postoperative strength and functional outcomes of the affected site have been evaluated. The postoperative strength assessment was conducted by measuring knee extension strength [23, 28]. However, considering the RF muscle is biarticular [3], hip flexion should also be assessed for a more comprehensive evaluation. In addition to strength testing, a functional task, such as the single-leg hop for distance (SLH), should likewise be incorporated to determine lower extremity function [16].

The aim of this study was to quantify midterm results after surgical refixation of acute PRFA. Besides the assessment of the clinical outcome and sports activity level, the functional results were evaluated using an isokinetic dynamometer and the SLH of the affected vs. the unaffected leg. The proposed hypothesis was that surgical refixation leads to high return to competition (RTC) rates without significant strength loss compared to the contralateral side.

## Materials and methods

**Fig. 1:**
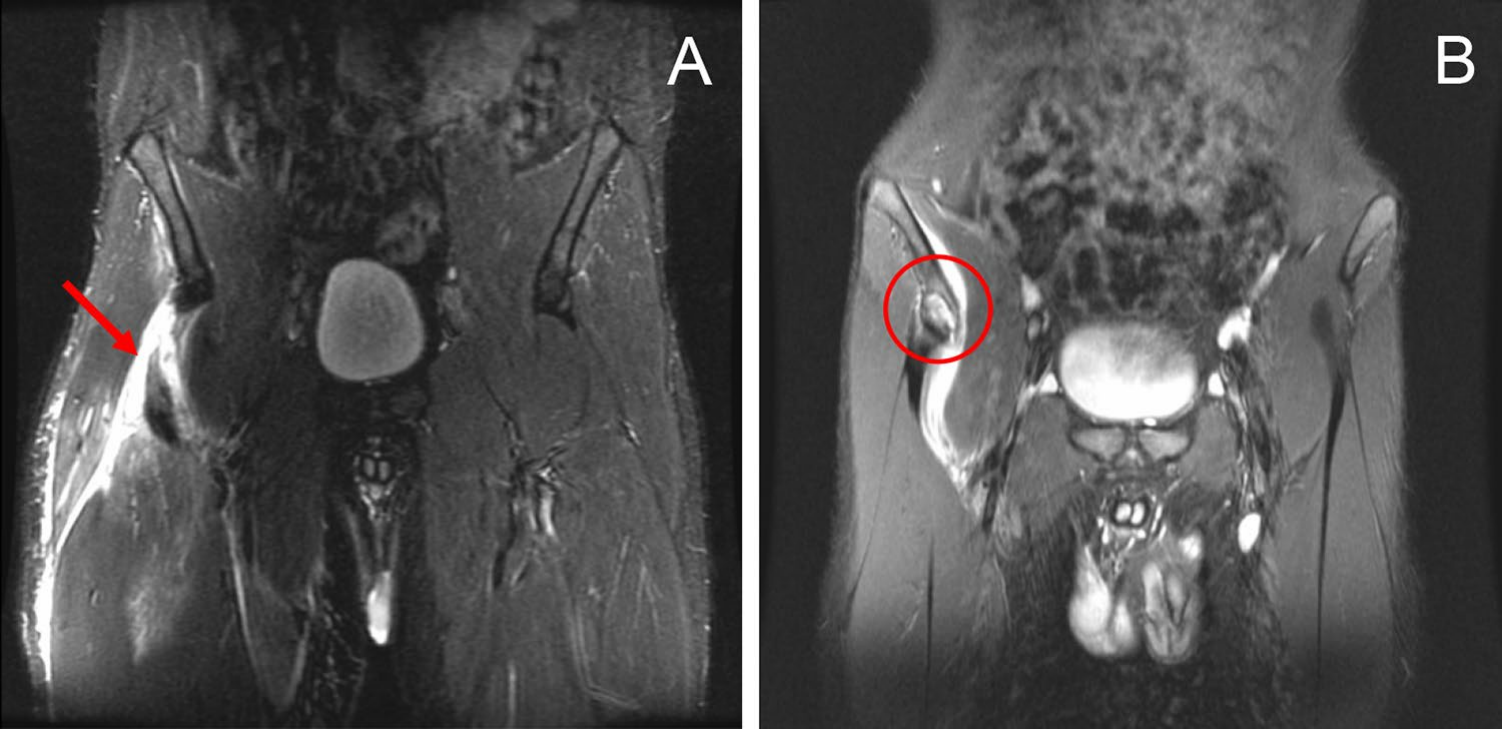
T2-weighed coronary MRI showing proximal avulsion of the rectus femoris which may appear tendinous (**A**) or bony (**B**)

Patients who underwent open surgical repair of PRFA between January 2012 and November 2019 were included for retrospective review. Patients were included if an acute (< 6 weeks since trauma) PRFA was confirmed by clinical examination and magnetic resonance imaging (MRI) (Fig. 1). Indication for early operative intervention was given in patients with both demanding physical requirements and complete or partial (i.e., subtotal), tendinous or bony PRFA who presented with pain, loss of strength in hip flexion and knee extension, as well as impaired function of the affected lower limb. Conservative treatment was reserved for those with low physical demand and patients who would not adhere to the postoperative rehabilitation protocol. Exclusion criteria were chronic injuries (> 6 weeks since initial trauma) and recurrent PRFA injuries where initial surgery was performed elsewhere.

**Fig. 2:**
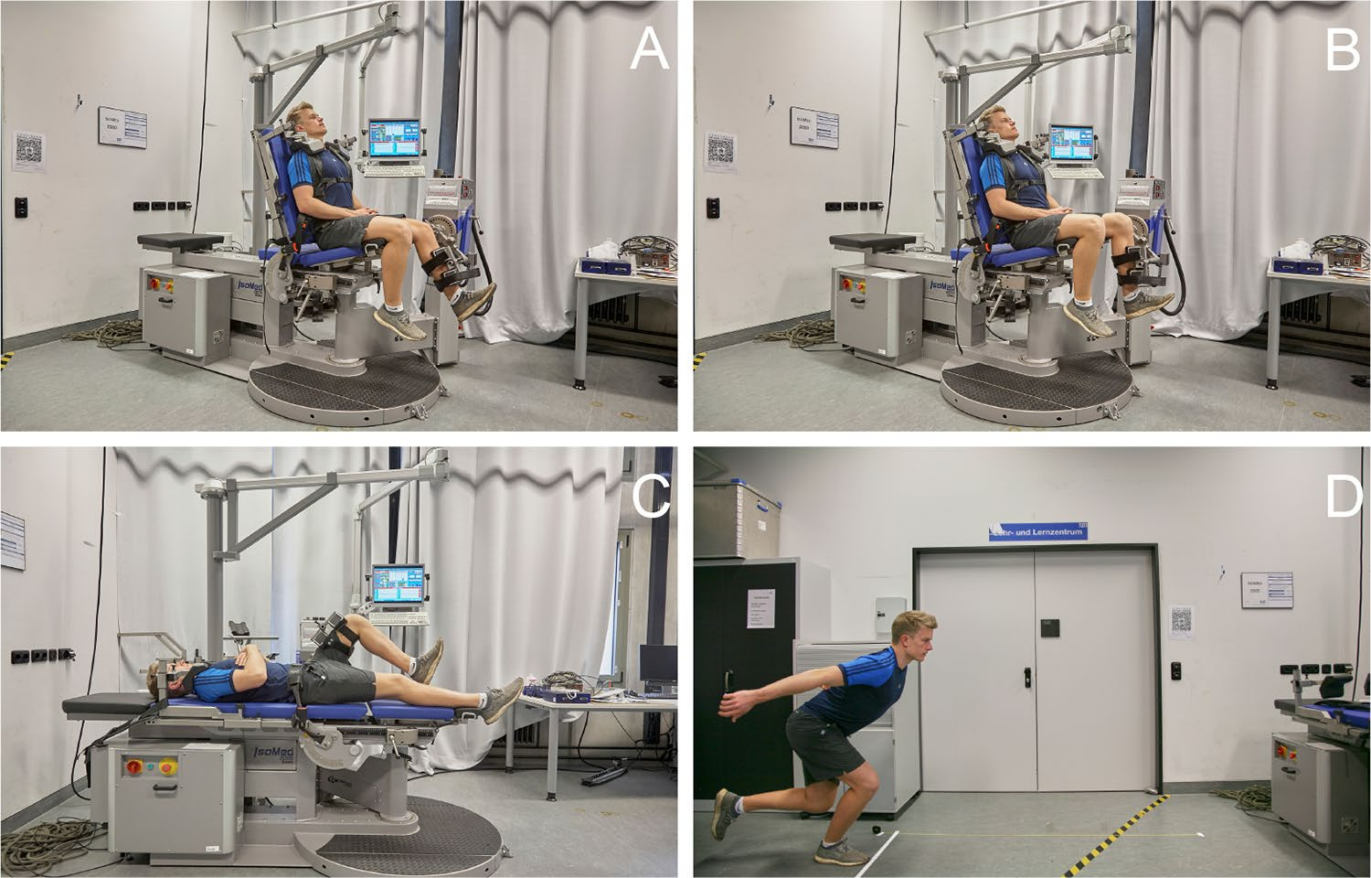
Postoperative strength of the affected limb was evaluated by measuring isometric knee extension (**A**) and knee flexion (**B**) at 60° and hip flexion at 60° (**C**). In addition, the single-leg hop for distance was performed at follow-up to assess lower extremity function (**D**)

Patients were invited for follow-up at our sports injury prevention center. Patient-reported outcome measures (PROMs) such as, the International Hip Outcome Tool-33 (iHot-33), Hip and Groin Outcome Score (HAGOS) subscales (symptoms, pain, function in daily living, function in sport and recreation, participation in physical activities and hip and/or groin-related quality of life), and the Harris Hip Score (HHS) were collected (minimum 12 months postoperatively) to quantify subjective function. Pain was assessed by the Visual Analog Scale (VAS) and postoperative sports participation was determined by the Tegner Activity Scale (TAS). Moreover, questions to obtain RTC rates and time frames, the extent to which return to work was possible, the level of satisfaction with the postoperative result, as well as information on postoperative complications with a special focus on injury recurrence, were asked. Objective measures such as, range of motion (ROM) of the hip and knee, thigh circumference (10 and 15 cm above the joint line of the knee), and heel-to-buttock distance were compiled to detect potential loss of muscle size or reduced flexibility. Then, following a general warm-up, the SLH as well as isometric knee extension, knee flexion, and hip flexion strength tests were performed. The SLH (Fig. 2D) is commonly used as a postoperative functional performance test following injuries to the lower extremity such as, anterior cruciate ligament tears, achilles tendon ruptures, and traumatic meniscus tears [5, 7, 13, 17]. An isokinetic dynamometer (IsoMed^®^ 2000, D&R Ferstl GmbH, Hemau, Germany) was used to evaluate unilateral isometric strength of the affected and unaffected sides. Specifically, maximum voluntary isometric contraction (MVIC) in single-joint knee extension and flexion, as well as hip flexion, was measured. MVIC was measured as peak torque in Newton meters (N*m) after a standardized warm-up session intended to activate the cardiovascular system and a mock practice session using the test equipment and setup. To assess knee extension and flexion, subjects were first seated in an upright position and secured by shoulder pads and hip and shoulder belts. The rotational dynamometer was calibrated for each subject. Two adjustable straps were used to position the leg at the pad of the lever arm. To assess knee extension strength, the subjects were asked to extend the knee, held in 60° of flexion, against the measuring pad at the front of the shin, using maximum quadriceps contraction for 5 s (Fig. 2A). In the same position, knee flexion strength was measured by asking the subject to pull against the measuring pad with maximum hamstring muscle contraction for the same amount of time (Fig. 2B) [20]. Hip flexion strength was measured in the supine position with hip flexion fixed at 60° (Fig. 2C). MVIC was measured three times for each muscle group with three minutes of rest in between each repetition. The highest value of maximum isometric torque measured was used for data analysis. The starting leg and order in which each muscle group was tested was randomized [11, 20]. Peak torque measures of knee flexion and extension were used to determine the hamstring to quadriceps ratio (H:Q $=\frac{\mathrm{peak hamstrings torque}}{\mathrm{peak quadriceps torque}} \times 100\mathrm{\%}$) [24]. Finally, all measurements were taken for the injured and uninjured limbs whereby a limb symmetry index (LSI) was calculated (LSI $=\frac{\mathrm{injured leg}}{\mathrm{uninjured leg}} \times 100$) [2].

The study was approved by the ethics committee of the Technical University of Munich (reference: 317/20 S) and conducted according to the Declaration of Helsinki. All participants gave their written informed consent.

### Surgical technique

**Fig. 3:**
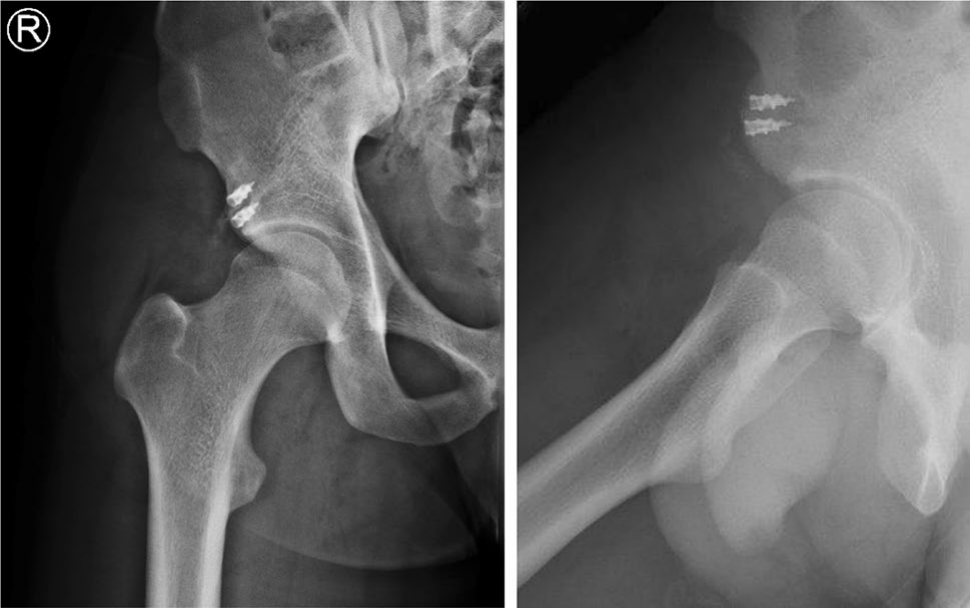
Postoperative radiographs. The antero-posterior and false-profile X-rays of the right hip show correct anchor placement at the anterior inferior iliac spine (straight head was reduced to superior anchor) and the superior acetabular ridge (reflected head was reduced to inferior anchor) with two double-loaded 5.5-mm titanium suture anchors (Corkscrew®, Arthrex, Naples, USA)

All patients were operated on at a single institution by experienced orthopaedic sports medicine surgeons. Procedures were performed with the patient in the supine position. A 7-cm longitudinal skin incision was made 2 cm distal to the anterior superior iliac crest and the rectus fascia was subsequently split along its fibers. After lateral retraction of the tensor fascia lata, identification and careful protection of the lateral cutaneous femoral nerve, and medial retraction of the iliacus muscle, the RF tendon was identified. The tendon was released from adhesions and scar tissue was debrided. The RF insertion—the anterior inferior iliac spine and superior acetabular ridge—was debrided and the anatomical footprint was decorticated to facilitate healing. Depending on the footprint and rupture extent, one to two double-loaded 5.5-mm titanium suture anchors (Corkscrew^®^, Arthrex, Naples, USA) were used for tendon refixation (Fig. 3). The tendon was firmly sutured with Krackow stitches from one strand and reduced by pulling on the second strand. Both sutures were tied on top of the reduced tendon. In cases with bony avulsions (Fig. 1B) and adequate bone stock of the fragment, refixation was performed by drilling holes into the fragment through which Krackow stitches were applied, linking the fragment and the tendon. As in tendinous ruptures, the fragment-tendon complex was reduced by firmly pulling on the second string and tying the sutures after the anatomic reinsertion was achieved. Finally, the wound was irrigated and closed.

### Postoperative rehabilitation

Postoperatively, the operated leg was secured in a Newport^®^ Easy hip brace (Ormed, Freiburg, Germany) with hip flexion limited to 30° and weight bearing was not permitted in the first 4 postoperative weeks. Physiotherapy started on the first postoperative day with passive flexion with patients receiving physiotherapy treatments 2–3 times per week. After 4 weeks, 20-kg weight bearing was approved for 2 weeks before full weight bearing and a slow increase in active muscle exercises were encouraged 6 weeks postoperatively.

### Statistical analysis

Data were analyzed using SPSS 26.0 (IBM-SPSS, New York, USA). Categorical variables are presented as count and percentage. Normal distribution of the collected continuous variables was assessed by the Shapiro–Wilk test and graphically confirmed. Normally distributed continuous variables are shown as mean ± standard deviation. Non-normally distributed continuous variables are shown as median (interquartile range). For group comparison of continuous variables, the Wilcoxon-test or paired *t*-test was applied. Statistical significance was set at a *p* value of < 0.05. Due to the rarity of the injury, all eligible patients were included and a power analysis was not carried out.

## Results

### Demographics

Sixteen out of 20 patients (80%) were available for retrospective analysis, of which 13 participated in the physical examination and strength assessment. The remaining 3 responded only to the survey. The majority of all injuries (87.5%) occurred during soccer—specifically while performing a shooting motion (69.6%). One soccer player also suffered a PRFA while jogging outdoors outside of soccer practice. A triathlete sustained his injury during a fall with hyperextension of the hip.

The mean follow-up was 44.8 ± 28.9 months (range 12–104). Three patients (15%) were unavailable for follow-up and one patient (5%) did not consent to participate and was, therefore, excluded from evaluation. The patients’ demographics are shown in Table 1.

**Table 1:** Patients’ demographics

	Number of patients
Number of patients (*N*)	16
Sex (male/female)	16/0 (100% male)
BMI (kg/m^2^)	24.9 ± 3.0
Injured side (right side/left side)	12/4 (75% right side)
Age at time of surgery (years)	27.6 ± 11.5
Time from trauma to surgery (days)	18.4 ± 8.5
Follow-up (months)	44.8 ± 28.9

Normally distributed continuous variables are shown as mean ± standard deviation, categorical variables are shown as percentages

### Patient-reported outcome measures

At follow-up, the patient-reported outcome by means of HHS, HAGOS subscales, iHOT-33, VAS and TAS was excellent (Table 2).

**Table 2:** Patient-reported outcome measures

Patient-reported outcome measures	Results
Harris Hip Score	100^a^ (96–100)
HAGOS	
Symptoms	94.6^a^ (89.3–100)
Pain	97.5^a^ (92.5–100)
Function in daily living	100^a^ (95–100)
Function in sport and recreation	98.4^a^ (87.5–100)
Participation in physical activities	100^a^ (87.5–100)
Quality of life	83.1 ± 15.6
iHOT-33	95.1^a^ (81.6–99.8)
Tegner Activity Scale	9^a^ (7–9)
Visual Analog Scale	0^a^ (0–0.5)

Normally distributed continuous variables are shown as mean ± standard deviation. Non-normally distributed continuous variables are shown as median

*HAGOS* Hip and Groin Outcome Score, *iHOT-33* International Hip Outcome Tool.

^a^Values are median

The majority of patients were “very satisfied” (*N* = 9, 56.3%) or “satisfied” (*N* = 6, 37.5%) with the postoperative result. Only one patient (6.3%) responded as being very “unsatisfied” due to continuous pain. Return to work was fully achieved by every patient. No postoperative complications or revision surgeries were reported.

### Return to sport

Out of the 16 patients, 15 were soccer players (of which 14 were active members of a team) and 1 patient was a triathlete. Of the 14 soccer players who were members of a team, 12 (85.7%) returned to competition at a mean time of 10.1 ± 3.6 months: 10 reported complete return to their previous performance level, two of whom reported minor constraints. The remaining two team-associated soccer athletes reported a reduction in performance level when compared to their pre-injury level. One patient stopped playing soccer due to the injury and the other patient was unable to return to soccer due to the COVID-19 pandemic, however, played competitive tennis 12 months postoperatively. Finally, the triathlete respondent achieved RTC 12 months following surgery.

### Functional outcome

Knee and hip ROM, heel-to-buttock distance, and thigh circumference (as an average of two measures: 10 and 15 cm superior to the joint line of the knee), were comparable between the operated and the contralateral legs. Furthermore, no significant strength or functional impediment of the operated leg was detected using MVIC of knee flexion and extension, hip flexion, and the SLH. Consequently, H:Q was similar between both legs and LSIs were favourable (Table 3).

**Table 3:** Results of the strength and functional assessment

	Operated leg	Contralateral leg	*p* value
ROM knee flexion (degrees)	141.2 ± 11.9	142.3 ± 10.7	n.s.
ROM knee extension (degrees)	+ 8.9 ± 5.1	+ 9.2 ± 4.9	n.s.
ROM hip flexion (degrees)	128.9 ± 16.1	132.7 ± 13.2	n.s.
ROM hip extension (degrees)	30^a^ (27.5–30)	30^a^ (27.5–30)	n.s.
Heel-to-buttock distance (cm)	7.3 ± 7.8	6.4 ± 7.4	n.s.
Thigh circumference (cm)	45.8 ± 4.4	46.0 ± 4.4	n.s.
Single-leg hop distance (cm)	173.5 ± 21.1	171.1 ± 25.3	n.s.
MVIC knee extension (N*m)	244.5 ± 60.9	247.4 ± 66.5	n.s.
MCIV knee flexion (N*m)	111.1 ± 18.8	111.2 ± 26.5	n.s.
MVIC hip flexion (N*m)	106.7^a^ (90.8–117.0)	110.6^a^ (96.0–120.3)	n.s.
H:Q (%)	47.4^a^ (42.6–52.0)	45.8^a^ (42.1–48.9)	n.s.
LSI			
SLH	100^a^ (98.1–103.0)		n.a.
Knee extension	99.3 ± 9.8		n.a.
Knee flexion	102.1 ± 12.9		n.a.
Hip flexion	95.8^a^ (85.6–107.7)		n.a.

Normally distributed continuous variables are shown as mean ± standard deviation. Non-normally distributed continuous variables are shown as median

*H:Q* hamstring to quadriceps ratio, *LSI* limb symmetry index, *MVIC* maximum voluntary isometric contraction, *ROM* range of motion, *SLH* single-leg hop for distance

^a^Values are median

## Discussion

The findings of this study support the hypothesis that surgical refixation after acute PRFA yields favourable results in a highly active population without functional restrictions compared to the contralateral leg. Out of the 16 patients included in this study, 15 were competitive athletes of which 14 (93.3%) returned to competition following surgery. Similar findings were reported in other studies following surgical anchor repair [15, 21, 26, 32]. Lempainen et al. [26] reported 19 cases in 18 professional soccer players with PRFA. All players returned to their pre-injury level of play. In two players, however, a persistent local loss of normal sensibility due to an injury to the lateral femoral cutaneous nerve branches was reported, which was not seen in the present cohort. Ueblacker et al. [32] assessed RTP and RTC data in four professional soccer players with a mean time from injury to surgery of 60 ± 88 days. In their cohort, RTP was achieved 111 ± 15 days and RTC was achieved 140 ± 23 days after surgery with all players returning to their pre-injury level and no reported complications. Irmola et al. [21] reported comparable results in a case series with four professional soccer players and one national-level hurdler with a median return to pre-injury level time period of 9 months (range 5–10 months). Garcia et al. [15] presented ten professional soccer players undergoing surgery for different proximal RF injuries. Four athletes suffered from a PRFA and were treated with suture anchor refixation of the avulsed tendon. All ten soccer players returned to official match activities 3.8 ± 0.8 months postoperatively. It was reported that all athletes returned to their previous level of play without any injury recurrence or pain. A sub-group analysis by different injury patterns and concomitant repair technique was not performed.

As an alternative to suture anchor refixation after PRFA, a technique for proximal tenodesis through a side-to-side suture to the surrounding muscles, primarily the vastus lateralis, has recently been proposed [23, 28]. Sonnery-Cottet et al. [28] reported an excellent outcome with a fast return to previous level of play at 15.8 ± 2.6 weeks postoperatively in a case series of five professional soccer players. However, isokinetic strength testing of knee extension and flexion 3 months postoperatively revealed no strength deficit in only two players, a low-to-moderate strength deficit in one player, and a moderate-to-high strength deficit in two players. This may be due to the short follow-up time, but is in contrast to our findings. Furthermore, extrapolation of their data to acute PRFA injuries may be limited as four out of five patients in their cohort had at least two prior RF injuries at the musculotendinous junction. Kayani et al. [23] prospectively compared the outcome of primary tenodesis vs. that of proximal refixation for the treatment of PRFA injuries. They reported a significantly faster return to pre-injury level and reduced risk of recurrence for the group treated with primary tenodesis (12.4 ± 1.6 vs. 15.8 ± 2.2 weeks, *p* < 0.001; 0% vs. 16%, *p* < 0.001). The high recurrence rate of 16% found in the group treated with surgical refixation, however, cannot be supported by the current study.

Strength assessments using an isokinetic dynamometer and functional assessments via SLH play an integral role in quantifying results after surgical treatment of various injuries to the lower extremity such as, proximal hamstring ruptures, achilles tendon ruptures, anterior cruciate ligament tears, and traumatic meniscus tears [1, 5–7, 13, 17, 31]. In our findings, no significant differences were noted between the operated and contralateral legs. The H:Q in our cohort (operated leg median 47.4; contralateral leg median 45.8) is comparable to the mean H:Q value of 49 observed in healthy male soccer players and considerably lower than the value that has been observed in a non-athletic population (60.6) [27, 34].

While return to sport/play/match and competition rates are reported by most studies regarding the surgical treatment of PRFA injuries [15, 21, 23, 26, 28, 32], quadriceps strength measurements were only conducted by two studies [23, 28] and hamstring strength was assessed only by one study [28]. To our knowledge, this is the first study to additionally assess hip flexion strength - an important function of the RF muscle - following proximal RF tendon refixation [3]. In addition, a functional test, such as the SLH, was performed to assess lower limb function following surgical treatment. The results of the SLH for the operated leg (mean 173.5 cm) and contralateral leg (mean 171.1 cm) showed no significant difference (*p* = 0.384), and are considerably higher than normative data collected in male high school athletes (mean 155 cm) [10].

PRFA injuries are often treated non-operatively, with mixed results. Gamradt et al. [14] retrospectively reviewed 11 cases of conservatively treated PRFA injuries in National Football League (NFL) athletes. They reported a mean RTP after 69.2 days, ranging from 21 to 208 days. Hsu et al. [18] also reported two cases of conservatively treated PRFA injuries in the NFL with a rapid recovery. However, one of the two athletes could no longer meet the performance standards of the team following the injury.

A recent meta-analysis by Dalal et al. [9] on conservative vs. operative treatments of PRFA injuries found similar results between the groups with a slightly higher return to sport rate for the surgically treated group (95%) compared to the conservatively treated group (92.7%), without statistical significance (*p* = 0.93). In addition, an earlier return to sport was observed for injuries that underwent surgery immediately vs. injuries that underwent surgery after failed initial conservative treatment (112.6 days vs. 204.6 days). This discrepancy supports the assumption that early surgical intervention may be favourable in young patients with high athletic demands. Straw et al. [30] reported an example of a semiprofessional soccer player with a chronic PRFA (at the myotendinous junction) that failed conservative treatment. Despite a 12-month long rehabilitation program, the affected quadriceps retained only 66% of the concentric power of the contralateral side. The patient opted for surgery (by direct suture) and recovered almost full concentric quadriceps power 6 months postoperatively.

The strengths of the present study are as follows: this was a single-center cohort study, patients were included using objective inclusion and exclusion criteria, MRI scans were used preoperatively to confirm diagnoses, all patients were treated using a standardized operative technique, postoperative rehabilitation was standardized for all study patients, and a comprehensive range of outcomes such as, functional outcome scores, RTC data, as well as strength and function assessments, were gathered at an average follow-up of 44.8 months.

This study has several limitations that must be considered when interpreting the findings. First, as surgical repair for PRFA injuries is an intervention choice usually indicated for highly active athletes, conservatively treated patients were not included in this study. Therefore, a comparative control group was not included and a comparison between surgical repair vs. conservative treatment outcomes could not be made. Second, all patients studied were highly active athletes which, despite creating a homogenous group, limits the generalizability of these data to less active patients.

The findings of this study will contribute to a more informed discussion about the surgical treatment of PRFA injuries and the expected postoperative result in highly active patients.

## Conclusion

Surgical refixation of acute proximal rectus femoris tendon avulsions yields excellent return to competition rates in a highly active cohort without significant strength or functional deficits when compared to the contralateral leg. Patients report little to no pain at short-to-midterm follow-up with zero surgical complications reported.

## Abbreviations

H:Q: Hamstring to quadriceps ratio
HAGOS: Hip and Groin Outcome Score
HHS: Harris Hip Score
iHot-33: International Hip Outcome Tool-33
LSI: Limb symmetry index
MRI: Magnetic resonance imaging
MVIC: Maximum voluntary isometric contraction
PRFA: Proximal rectus femoris avulsion
PROMs: Patient-reported outcome measures
RF: Rectus femoris
ROM: Range of motion
RTC: Return to competition
RTP: Return to play
SLH: Single-leg hop for distance
TAS: Tegner Activity Scale
VAS: Visual Analog Scale
